# Changes in Northern Elephant Seal Skeletal Muscle Following Thirty Days of Fasting and Reduced Activity

**DOI:** 10.3389/fphys.2020.564555

**Published:** 2020-10-06

**Authors:** Traver J. Wright, Randall W. Davis, Rachel R. Holser, Luis A. Hückstädt, Christopher P. Danesi, Craig Porter, Steven G. Widen, Terrie M. Williams, Daniel P. Costa, Melinda Sheffield-Moore

**Affiliations:** ^1^Department of Health and Kinesiology, Texas A&M University, College Station, TX, United States; ^2^Department of Internal Medicine, University of Texas Medical Branch, Galveston, TX, United States; ^3^Department of Marine Biology, Texas A&M University, Galveston, TX, United States; ^4^Ecology and Evolutionary Biology, University of California, Santa Cruz, Santa Cruz, CA, United States; ^5^Department of Pediatrics, University of Arkansas for Medical Sciences, Little Rock, AR, United States; ^6^Department of Biochemistry and Molecular Biology, University of Texas Medical Branch, Galveston, TX, United States

**Keywords:** muscle atrophy, fasting, skeletal muscle, elephant seal, lipid metabolism

## Abstract

Northern elephant seals (NES, *Mirounga angustirostris*) undergo an annual molt during which they spend ∼40 days fasting on land with reduced activity and lose approximately one-quarter of their body mass. Reduced activity and muscle load in stereotypic terrestrial mammalian models results in decreased muscle mass and capacity for force production and aerobic metabolism. However, the majority of lost mass in fasting female NES is from fat while muscle mass is largely preserved. Although muscle mass is preserved, potential changes to the metabolic and contractile capacity are unknown. To assess potential changes in NES skeletal muscle during molt, we collected muscle biopsies from 6 adult female NES before the molt and after ∼30 days at the end of the molt. Skeletal muscle was assessed for respiratory capacity using high resolution respirometry, and RNA was extracted to assess changes in gene expression. Despite a month of reduced activity, fasting, and weight loss, skeletal muscle respiratory capacity was preserved with no change in OXPHOS respiratory capacity. Molt was associated with 162 upregulated genes including those favoring lipid metabolism. We identified 172 downregulated genes including those coding for ribosomal proteins and genes associated with skeletal muscle force transduction and glucose metabolism. Following ∼30 days of molt, NES skeletal muscle metabolic capacity is preserved although mechanotransduction may be compromised. In the absence of exercise stimulus, fasting-induced shifts in muscle metabolism may stimulate pathways associated with preserving the mass and metabolic capacity of slow oxidative muscle.

## Introduction

Northern elephant seals (NES) have an annual cycle that includes months at sea foraging and acquiring body fat, punctuated by extended fasting periods while hauled out on land for molting or pupping/breeding (Fowler et al., 2018). When molting, these seals typically spend ∼40 days on land with the combined catabolic stimuli to skeletal muscle of reduced activity and fasting. During this time, adult females lose approximately one-quarter of their body mass, with 95% of their energy derived from the oxidation of endogenous fat stores. Despite the profound loss of body fat and reduced activity, skeletal muscle mass is preserved (Worthy et al., 1992). Following these extended periods of reduced activity, seals promptly return to physical activity at sea including long migrations and extended foraging dives. Although skeletal muscle mass is largely preserved during molt, little is known about the functional capacity of that muscle after prolonged periods of reduced activity. The lifestyle of NESs makes them a novel model species for studying adaptations in skeletal muscle to unloading and energy restriction.

Skeletal muscle is a highly plastic tissue that readily adapts to physical demands including an anabolic response to increased muscle load (e.g., exercise and increased demand for force production) and a catabolic response to muscle unloading (e.g., inactivity, bedrest, joint immobilization, and spaceflight) (for review see Seene et al., 2012; Frontera and Ochala, 2015; Mukund and Subramaniam, 2019). Changes in response to anabolic and catabolic stimuli alter not only the skeletal muscle mass, but also the metabolic infrastructure and respiratory capacity. In addition, structural integrity and force transduction capacity is altered by regulating expression of contractile proteins and components of the extracellular matrix (ECM) including collagen.

Our current understanding of the regulation of mammalian skeletal muscle mass and metabolism is based largely on a few species of terrestrial omnivores including mice, rats, and humans, which limits the comparative implications of those findings. Although the mechanisms regulating skeletal muscle hypertrophy and atrophy are not fully understood, long-term maintenance of muscle mass requires a balance of anabolic protein synthesis and catabolic proteolysis (Glass, 2003, 2005). The stimuli and mechanisms regulating this balance are not the same for all skeletal muscle fiber types because different stimuli initiate signaling cascades that affect slow oxidative type I fibers or fast glycolytic type II fiber types unequally (Ciciliot et al., 2013; Wang and Pessin, 2013; Cannavino et al., 2015; Dowling et al., 2016). This differential effect on slow and fast fiber types can result in a shift in fiber type proportion depending on the atrophic stimulus with a trend toward greater relative proportion of slow twitch fibers typically associated with fasting and aging, and a trend favoring fast twitch fibers typically associated with hypoxia and muscle disuse (Dowling et al., 2016).

Although skeletal muscle mass is preserved in molting NES despite prolonged fasting and reduced activity, potential changes in skeletal muscle metabolic and functional capacity have not been examined. In the current study, we sought to understand the regulatory mechanisms in NES skeletal muscle that protect skeletal muscle mass during the annual molt. We assessed changes in gene expression and metabolic capacity of female NES skeletal muscle soon after arrival on shore and after ∼30 days of reduced activity and fasting.

## Materials and Methods

### Animal Sampling

Animal sampling was conducted under NMFS permit number 19108, and was approved by the institutional animal care and use committee of the University of California, Santa Cruz (IACUC protocol Costa1409). Skeletal muscle tissue was collected during the annual spring molt at Año Nuevo State Park, San Mateo County, CA, United States. Samples were collected to assess changes in the respiratory capacity and gene expression in adult female elephant seal skeletal muscle early and late during the molt period when seals remain on land with reduced activity and fasting. Study animals were subjects of a separate parallel study and their location at sea was monitored by satellite tag prior to the molt haul-out. Baseline pre-molt muscle biopsies were taken within 5 days of arrival on the beach as determined by satellite tracking and visual confirmation. Subsequent post-molt samples were taken approximately 1 month after the initial sample (average: 34 days; range: 28–37 days). A total of 19 skeletal muscle biopsies were taken from 11 adult female NES. Both pre and post-molt samples were collected for a total of 7 animals. When conditions allowed, body mass was determined using a hanging scale suspended from a tripod as previously described (Robinson et al., 2012). Paired pre and post-molting body mass was collected for 4 of the 7 animals.

Animals were initially immobilized by intramuscular injection of Telazol (1 mg kg^–1^) and sedation was maintained with ketamine and diazepam injections via a spinal needle inserted into the extradural vein (MWI Animal Health) as previously described (Robinson et al., 2012). The biopsy site (approximately two thirds of body length from head, adjacent to midline) was sterilized with Betadine, and a local anesthetic (1% Lidocaine) was administered before making a small (approximately 1 cm) incision in the skin. A 6 mm Integra Miltex dermal biopsy punch (Thermo Fisher Scientific) was used to take a core of both blubber and muscle from the site, and a 6 mm Bergström biopsy cannula was then inserted through the blubber into the *longissimus dorsi* muscle to collect a second muscle biopsy. For each biopsy approximately 50 mg of muscle tissue was collected. Tissue from the first biopsy was immediately placed in a 2 ml vial of chilled biopsy preservation buffer (BIOPS; pH 7.1) containing 10 mM Ca^2+^-EGTA, 0.1 μM free calcium, 20 mM taurine, 50 mM K-MES, 0.5 mM DTT, 6.56 mM MgCL_2_, 5.77 mM ATP, and 15 mM phosphocreatine. BIOPS samples were kept on ice, and returned to the lab to be processed the following day or shipped overnight for processing within 24 h of collection. A second muscle biopsy was preserved in 2 ml of RNAlater (Sigma, R0901) that was chilled on ice or refrigerated for 48 h, before being frozen for later RNA extraction and gene expression analysis.

### High Resolution Respirometry

#### Tissue Preparation

Tissue preparation and permeabilization for high resolution respirometry was performed in a 6 well culture dish at 4°C. In 2 ml of chilled BIOPS buffer, muscle fiber bundles were teased apart using fine tip forceps under magnification to increase surface area. Following mechanical separation, fiber bundles were transferred to 2 ml of fresh BIOPS buffer with 50 μg ml^–1^ saponin for 20 min under gentle rocking to permeabilize the outer cell membrane and wash out intracellular substrates and adenylates. Permeabilized fiber bundles were then transferred to 2 ml of mitochondrial respiration buffer (MIRO5) containing 0.5 mM EGTA, 3 mM MgCl_2_, 60 mM lactobionic acid, 20 mM taurine, 10 mM KH_2_PO_4_, 20 mM HEPES, 110 mM sucrose, and 1 g/l fatty acid free BSA, and gently rocked for an additional 10 min to wash out any residual saponin. Fiber bundles were removed from the buffer solution with fine forceps and blotted with a Kimwipe to remove surface moisture before weighing on a micro-balance (Mettler Toledo AB204-S/PH; Mettler-Toledo, Columbus, OH, United States). Two to five milligram of tissue was transferred to the chamber for each respirometry protocol.

#### Respirometry

High-resolution tissue respirometry was performed using an Oxygraph-2K (O2K) respirometer (Oroboros Instruments, Innsbruck, Austria) according to previously described principles (Pesta and Gnaiger, 2012) using protocols adapted from previous studies (Porter et al., 2014, 2015). Paired pre and post-molt samples were viable for respirometry analysis in 6 of the 7 individuals with paired samples. Before each protocol, the oxygen electrode for each chamber was calibrated using ambient air. The MIRO5 respiratory buffer solution was maintained at 37°C throughout each protocol and stirred continuously at 750 rpm by a magnetic stirrer. Oxygen concentration was monitored with an attached computer using DatLab software (Oroboros Instruments, Innsbruck, Austria). Following temperature equilibration and oxygen calibration, muscle tissue samples (prepared as described above) were introduced into the chamber before partially sealing the chamber with a stopper. Oxygen was injected into the air space above the stirred buffer, and the increasing oxygen concentration was monitored. After the buffer oxygen concentration reached approximately 400 nmol/ml, the remaining gas was forced out, sealing the chamber. Oxygen concentration was maintained between 250 and 400 nmol/ml throughout each protocol in an attempt to prevent oxygen diffusion limiting tissue respiration. After establishing steady state baseline respiratory flux in the metabolic chamber with the absence of metabolic precursors, individual protocols were started with protocol specific stepwise titration of substrates, adenylates, and inhibitors. A new steady state oxygen consumption rate was established for each metabolic state before proceeding with titrations for the next metabolic state. Four separate substrate uncoupler inhibitor titration (SUIT) protocols were performed for each sample including A, B, CI, and CII (Table 1).

**TABLE 1:**
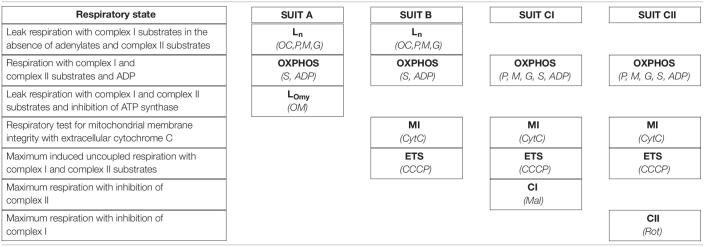
Stepwise titration of substrates and inhibitors utilized four distinct SUIT protocols (A, B, CI, CII) to achieve the following skeletal muscle respiratory states: maximum leak with complex I substrates and no adenylates (L_n_), OXPHOS respiratory capacity through complex I and II, leak respiratory capacity in the presence of complex I and II substrates with inhibition of ATP synthase (L_Omy_), uncoupled electron transport system respiratory capacity (ETS), and uncoupled maximum respiratory capacity through complex I (CI) and complex II (CII).

The substrate, uncoupler, and inhibitor concentrations used in each of the SUIT protocols were; 0.5 mM octanoylcarnitine (*OC*), 10 mM pyruvate (*P*), 0.5 mM malate (*M*), 10 mM glutamate (*G*), 10 mM succinate (*S*), 5 mM ADP (*ADP*), 10 μM cytochrome C (*CytC*), 5 μM oligomycin (*OM*), 0.5 μM CCCP (*CCCP*), 1 μM rotenone (*Rot*), and 10 mM malonate (*Mal*). The addition of cytochrome C was used as a test for mitochondrial membrane integrity, and was not used to induce an experimental respiratory state. An increase in respiratory flux of greater than 10% following cytochrome C titration was used as a threshold to indicate that compromised outer mitochondrial membranes invalidated the respirometry measures for that sample.

Using high resolution respirometry we assessed NES *longissimus dorsi* skeletal muscle oxygen consumption *ex vivo* in primary respiratory states including measures of leak respiration, OXPHOS, and ETS as well as max respiratory flux through respiratory complexes I (CI) and II (CII). Uncoupled ETS respiration reflects the maximum mitochondrial respiratory flux as a measure of mitochondrial density (Votion et al., 2012) of which the other respiratory states make up various portions. OXPHOS respiratory capacity represents the portion of ETS capacity composed of both the respiratory flux coupled to mitochondrial oxidative phosphorylation through ATP synthase and non-coupled leak respiration.

Steady state respiratory values were corrected for baseline respiratory chamber oxygen flux determined at the beginning of each protocol. Because the availability of lipid substrate was not limiting to OXPHOS respiration (absence of octanoylcarnitine in protocols CI and CII did not alter OXPHOS measures; *p* = 0.530), the OXPHOS measurements in protocols CI and CII were averaged together with protocols A and B for analysis. For respiratory states that were measured repeatedly for each sample due to overlapping protocol measures (i.e., L_n_, OXP, ETS), the average of those measures were analyzed for each time point. Respiratory states determined by inhibitory titration with only a single measure for each sample (i.e., L_Omy_, CI, CII) were standardized to the average OXPHOS for that sample by multiplying the fractional reduction from OXPHOS by the average OXPHOS for that sample. The difference between pre and post fasting respiratory measures was assessed using repeated measures *t*-tests. Respiratory ratios were calculated to assess the relative contribution of individual respiratory states to respiratory capacity. Substrate control ratios (SCR) were calculated to assess the contributions of complex I and complex II to total OXPHOS capacity (SCR_CI_: CI/OXPHOS and SCR_CII_: CII/OXPHOS). To assess the relative contribution of LEAK respiration to total OXPHOS respiration, we calculated the coupling control ratio (CCR: LEAK/OXPHOS). To assess the reserve respiratory capacity in the mitochondrial ETS system in excess of OXPHOS capacity, we calculated a flux control ratio (FCR: OXPHOS/ETS). Comparisons between pre and post-molt measures were assessed using paired *t*-tests with a significance threshold of *p* < 0.05.

### Mass-Loss Estimates of Molt Metabolism

In order to provide context to the measured respiratory capacities in this study, daily energy expenditure was estimated using the daily average loss of body mass based on the distribution of tissue loss (e.g., fat vs. lean tissue) and the energy derived from catabolism of each of those tissue categories. A previous study of similarly sized molting female NES showed that the lost mass was composed of 14% molted pelage. The remaining 86% was composed of 41% fat, 25% water, and 20% metabolizable fat-free mass (with fat-free mass composed of 5% dry matter and 15% additional water) (Worthy et al., 1992). Daily energy expenditure was estimated based on metabolic equivalents for fat of 9 kcal g^–1^ (37.7 kJ g^–1^) and fat-free mass of 1 kcal g^–1^ (4.2 kJ g^–1^) (Tataranni et al., 2003).

### Gene Expression

Skeletal muscle tissue samples preserved and frozen in RNAlater were homogenized and prepared for RNA extraction. Samples were flash-frozen in liquid nitrogen and pulverized using a mortar and pestle before mechanical homogenization with a rotary tissue grinder using an RNAqueous Total RNA Isolation Kit (Thermo Fisher Scientific). RNA samples were quantified using a Qubit fluorometer. Poly-A + RNA was enriched from ∼0.05 μg of total RNA and used as a template to generate sequencing libraries using the New England Biolab NEBNext Ultra RNA Library Prep Kit. Following the supplier’s protocol the RNA was fragmented at 94°C for 15 min. After cDNA synthesis, end repair and adapter ligation, libraries were amplified by 14 cycles of PCR. The indexed libraries were pooled and sequenced on three lanes of an Illumina HiSeq 1,500 High-output flow cell with the 50 base paired-end protocol.

The reads in fastq format were aligned to a reference genome using the splicing aware software STAR, version 2.5.3a_modified, using the ENCODE recommended parameters. The genome index was built with the NCBI Hawaiian monk seal (Neomonachus schauinslandi) reference genome, assembly GCA_00221575.1, with the associated NCBI annotation file, Release 100. Reads mapped to genes were quantified with the STAR –quantMode GeneCounts option (Dobin et al., 2013). The Hawaiian monk seal genome was utilized as the most complete and closest related genome available (for summary of read map alignment see Supplementary Table S1). The mapped reads had approximately 1% mismatch rate.

Because some samples gave poor sequencing results due to low quality input RNA as reflected by FastQC (Bioinformatics, 2015) analysis and read alignment results, we selected the 4 samples from each condition (pre-molt and post-molt) with the most unique reads (not paired) for further analysis. The read counts per gene for each sample were input into the DESeq2 (Love et al., 2014) differential expression program. Following the DESeq2 vignette, differentially expressed genes were called with an adjusted *p*-value cut-off of less than 0.05 and a log_2_ fold-change of ±1. Gene expression data has been deposited in NCBI’s Gene Expression Omnibus (Edgar et al., 2002) and is accessible by GEO accession number GSE151509^1^.

Pathway enrichment analyses of upregulated and downregulated gene sets were assessed against the KEGG 2019 Human pathway database using Enrichr (Chen et al., 2013; Kuleshov et al., 2016). Significantly altered metabolic pathways were identified by an adjusted *p* < 0.05 using the Fisher exact test and visualized based on an Enrichr combined score calculated by multiplying the log of the *p*-value from the Fisher exact test by the z-score of the deviation from the expected rank.

## Results

### Body Mass and Metabolism

We measured the body mass of female NES before and after the annual molt (Table 2). During this period, seals remain on shore for approximately 6 weeks with reduced activity and no access to food resulting in significant loss of body mass. Of the seven seals in our study, we were able to collect both pre and post-molt body mass from four. The initial body mass averaged 388 ± 50 kg, and they lost an average of 101 kg (3 kg day^–1^), equivalent to 26% of their initial mass during the molt (Table 2, white). This loss of body mass is consistent with previously reported values in similarly sized molting female NES (Worthy et al., 1992).

**TABLE 2:**
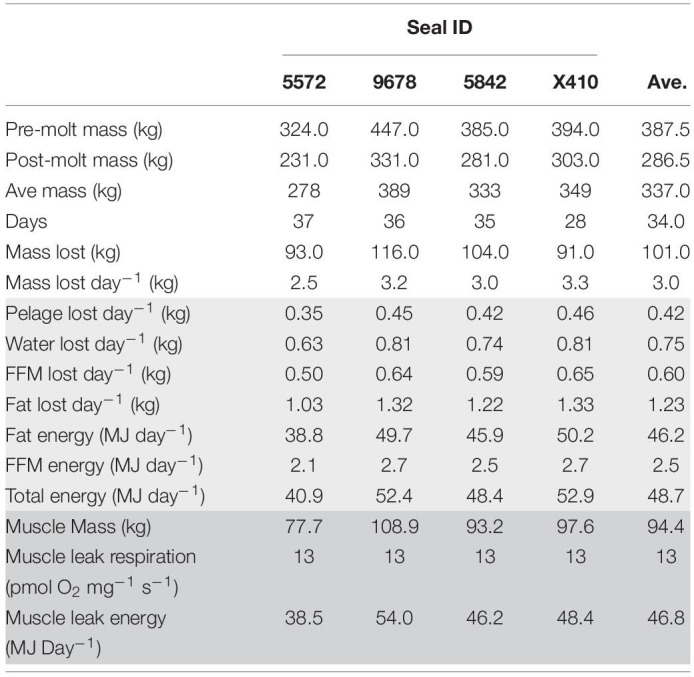
Measurements taken from four northern elephant seals at pre and post-molt timepoints. Direct measures are depicted in white.

*Additional estimated measures of distribution of lost mass (Worthy et al., 1992) and energy derived from those losses (Tataranni et al., 2003) are presented in light gray. Estimated total skeletal muscle leak respiration based on the lowest measures of leak respiratory capacity determined in this study and estimated muscle mass (Ponganis et al., 2011) are depicted in dark gray.*

Based on the loss of body mass and the distribution of catabolized tissue while fasting (see “Materials and Methods” section for detailed description), a loss of 3 kg of mass per day was apportioned as 0.42 kg of lost pelage, 0.75 kg of water, 0.60 kg of fat-free mass, and 1.23 kg of fat mass lost daily. The metabolic equivalent of catabolism of fat and fat-free mass gave an estimated metabolic rate of 48.7 MJ day^–1^ (11,670 kcal day^–1^) with 46.2 MJ day^–1^ (95%) derived from fat oxidation (Table 2, light gray). These estimates are consistent with an estimated metabolic rate of 46.5 MJ day^–1^ for similarly sized molting female NES determined using water turnover with an estimated 94% of energy derived from fat oxidation (Worthy et al., 1992).

**FIGURE 1:**
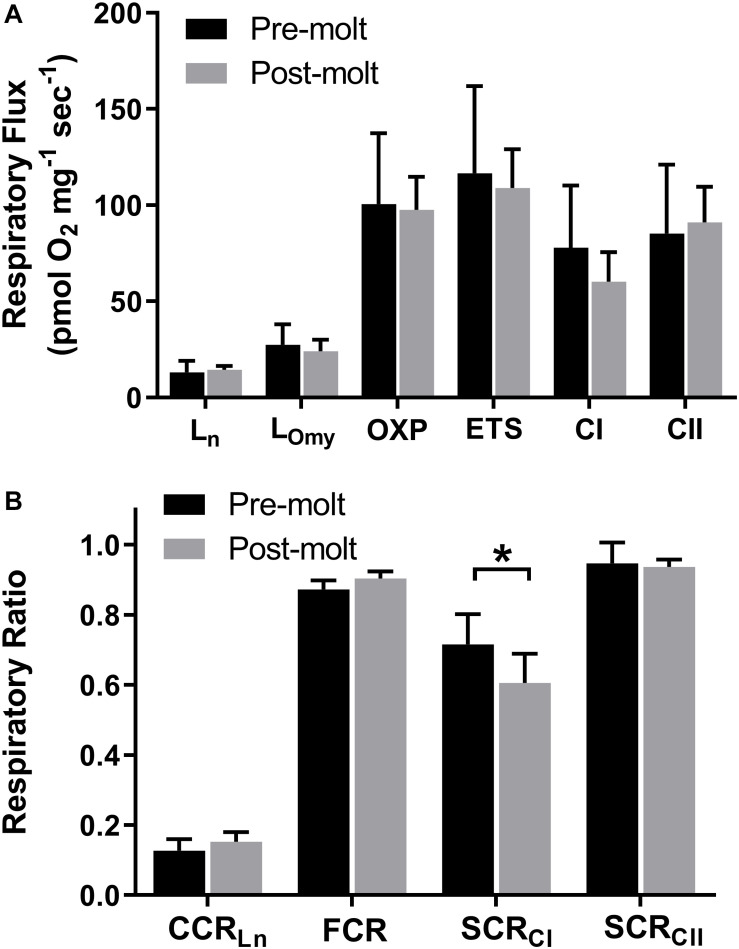
Northern elephant seal skeletal muscle respiratory flux before and after approximately 1 month during the yearly molt. Error bars represent standard deviation. High resolution respirometry was used to assess **(A)** respiratory states of leak (L_n_ and L_Omy_), OXPHOS (OXP), ETS, CI, and CII. **(B)** Ratios of respiratory rates were calculated to assess the relative contribution of leak respiration to OXPHOS (L_n_/OXPHOS = CCR_Ln_), the relative contribution of CI to OXPHOS (CI/OXPHOS = SCR_CI_), the relative contribution of CII to OXPHOS (CII/OXPHOS = SCR_CII_), and relative contribution of OXPHOS to ETS (OXPHOS/ETS = FCR). Statistical significance (*p* < 0.05) is denoted by *.

Respiratory flux based on energy metabolism was used to provide context for skeletal muscle leak respiration. Assuming that ∼95% of energy was derived from fat oxidation, an oxygen equivalent of 19.7 kJ LO_2_^–1^ (Schmidt-Nielsen, 1997) was used to estimate skeletal muscle metabolism based on respiratory flux. With 28% of elephant seal body mass composed of skeletal muscle (Ponganis et al., 2011) and an average body mass in our study of 337 kg (average of pre and post-molt body mass), the estimated skeletal muscle mass for seals in our study was 94.4 kg. Assuming the lowest measure of skeletal muscle leak respiration in this study (respiratory results below) of 13 pmol O_2_ mg^–1^ s^–1^ (17.5 ml O_2_ kg^–1^ min^–1^; Figure 1A and Supplementary Table S2), we estimate a skeletal muscle leak respiratory capacity of 46.8 MJ day^–1^ (Table 2), which is roughly equal to the estimated total daily energy expenditure of 48.7 MJ day^–1^.

### Respirometry

We assessed the aerobic respiratory capacity of *longissimus dorsi* muscle before and after ∼30 days of fasting and reduced activity for six of the seven seals. Oxygen consumption was measured at induced respiratory states of mitochondrial leak, OXPHOS, electron transport system, complex I (CI), and complex II (CII) respiration using high resolution respirometry. In addition, respiratory ratios were used to assess the relative contribution to OXPHOS respiration for CI (SCR_CI_), CII (SCR_CII_), and leak respiration (CCR_Ln_). As a measure of mitochondrial efficiency, the contribution of OXPHOS to ETS (FCR) was assessed with a lower value indicating ETS capacity in excess of OXPHOS respiratory capacity.

**FIGURE 2:**
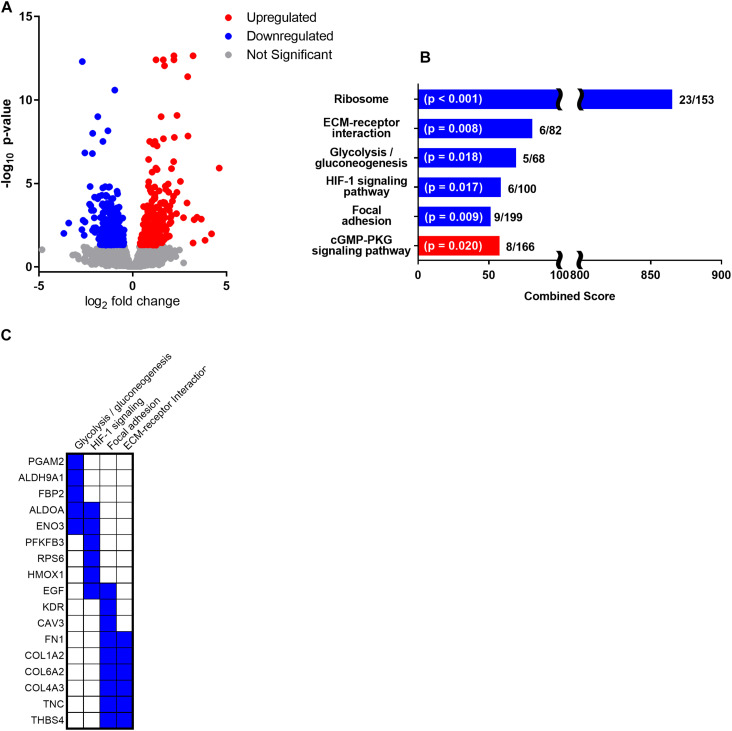
Following 1 month of reduced activity and fasting during the annual molt, NES skeletal muscle demonstrated differential gene expression in 642 genes including 313 downregulated genes and 328 upregulated genes (p_FDR_ < 0.05). **(A)** Volcano plot of fold change and *p*-value for upregulated and downregulated genes with significantly upregulated genes in red and significantly downregulated genes in blue. **(B)** Enrichr pathway analysis using significantly altered genes with ≥ 1 log_2_ fold change in gene expression (162 upregulated genes and 172 downregulated genes) was used to identify significantly upregulated (red) and 7 downregulated (blue) metabolic pathways. Bar length is presented as the Enrichr combined score calculated as the log of the *p*-value from the Fisher exact test multiplied by the z-score of the deviation from the expected rank. The *p*-value and ratio of the number of altered genes to the total number of pathway genes is noted for each. **(C)** There were 5 downregulated genes identified in the “Glycolysis/gluconeogenesis” pathway and 6 downregulated genes identified in the “HIF-1 signaling” pathway. The 9 downregulated genes in the “focal adhesion” pathway and 6 downregulated genes in the “ECM-receptor interaction” pathways had considerable overlap and were primarily related to skeletal muscle structural integrity.

There was no significant change in overall skeletal muscle respiratory capacity from pre to post-molt including measures of OXPHOS (*p* = 0.753) and total ETS (*p* = 0.553) (Figure 1A and Supplementary Table S2). Leak respiration as assessed both with complex I substrates and no adenylates (L_n_) and with CI and CII substrates with inhibition of ATP synthase (L_Omy_) did not change significantly from pre to post-molt (*p* = 0.602 and *p* = 0.298, respectively; Figure 1A and Supplementary Table S2). Although the two different measures of mitochondrial leak respiratory capacity did not change during molt, these two different methods of assessing leak respiration yielded significantly different results (*p* < 0.001). The contribution of leak (L_n_) to total OXPHOS capacity (CCR_Ln_) also did not change significantly from pre to post-molt (Figure 2B; *p* = 0.181).

Although the average respiratory flux supported by CI did not change significantly from pre to post-molt (*p* = 0.219; Figure 1A), SCR_CI_ decreased (*p* = 0.033; Figure 1B) indicating a reduced relative contribution of CI to total OXPHOS. The average respiratory flux supported by CII did not change from pre to post-molt (Figure 1A), and likewise, the relative contribution of CII to total OXPHOS did not change from pre to post-molt (Figure 1B; *p* = 0.756).

### Gene Expression

Gene expression analysis in NES skeletal muscle following 1 month of reduced activity and fasting identified differential expression in 642 genes including 313 downregulated and 328 upregulated genes (p_FDR_ < 0.05). Of those differentially expressed genes, 334 had a log_2_ fold change of less than -1 (172 downregulated genes; Supplementary Table S3A) or greater than 1 (162 upregulated; Supplementary Table S3B and Figure 2A). Enrichr pathway analysis was used to identify significantly altered pathways, and other significantly altered key regulatory genes are discussed in relation to their function.

#### Altered Signaling Pathways

Using Enrichr pathway analysis, the most profoundly downregulated pathway was “ribosome” gene expression (adj. *p* < 0.001; Figure 2B) including downregulation of 15 large ribosomal subunits (Supplementary Table S3A; *RPL*s as well as the one mitochondrial large ribosomal subunit, *MRPL10*) and 8 small ribosomal subunits (Supplementary Table S3A; RPSs) indicative of reduced skeletal muscle protein translation. Two downregulated pathways were primarily linked to skeletal muscle structural integrity and ECM including the KEGG pathways “ECM-receptor interaction” (adj. *p* = 0.008; Figure 2B) with 6 downregulated genes (Figure 2C) and “focal adhesion” (adj. *p* = 0.009; Figure 2B) with 9 downregulated genes (Figure 2C). The HIF-1 signaling pathway was significantly downregulated (adj. *p* = 0.017; Figure 2B) with 6 associated genes (Figure 2C). In addition, glycolysis/gluconeogenesis was significantly downregulated (adj. *p* = 0.018; Figure 2B) with 5 associated genes (Figure 2C).

Enrichr pathway analysis also identified a single significantly upregulated KEGG pathway as the “cGMP-PKG signaling pathway” (adj. *p* = 0.020; Figure 2B) with 8 upregulated genes (*ADRB2*, *ATP2A2*, *ATP1B4*, *IRS1*, *PRKG1*, *NOS3*, *MYLK2*).

## Discussion

### Overview

Molting NES experience the combined effects of both molting and prolonged fasting with reduced activity. Although there are significant physiological costs associated with the catastrophic molt experienced by NES, the molting process is not presumed in our analysis to directly influence skeletal muscle beyond competing for the limited systemic energy resources to replenishing skin and fur. In often-studied models of mammalian skeletal muscle, unloading typically causes quantitative and qualitative degradation including decreased mass, metabolic capacity, and structural integrity resulting in decreased force production (Seene et al., 2012). Although NES lose ∼25% of body mass during the annual molt (including ∼40 days of reduced activity and fasting), skeletal muscle mass is largely preserved (Worthy et al., 1992). Here we show that skeletal muscle metabolic capacity is also preserved during molt despite prolonged unloading. Previous studies have indicated that seals upregulate fat oxidation during fasting periods (Castellini et al., 1987; Castellini and Rea, 1992; Boily and Lavigne, 1995), and skeletal muscle gene expression analysis in this study is consistent with suppressed glucose oxidation and promotion of lipid oxidation.

### Skeletal Muscle Structural Integrity

Although skeletal muscle respiratory capacity and mass appear to be preserved during the seasonal molt, gene ontology suggests a downregulation of genes associated with mechanotransduction of force in skeletal muscle including focal adhesion, and ECM components. Increased mechanical loading in skeletal muscle typically results in an upregulation of collagen as well as proteoglycans and other components of the ECM (Kjaer, 2004; Ramachandran et al., 2019). Conversely, skeletal muscle unloading results in decreased transcriptional expression of ECM components including collagen genes (Savolainen et al., 1987; Seene et al., 2012), with as little as 48 h of unloading required for decreased gene expression (Urso et al., 2006). In addition to the downregulated “focal adhesion” and “ECM-receptor interaction” pathways, other key genes associated with structural integrity were downregulated including two additional collagen genes (*COL14A1*, *COL15A1*) as well as lumican (*LUM*), procollagen C-endopeptidase enhancer (*PCOLCE*), and matrix metallopeptidase 2 (*MMP2*). Although a number of related genes were downregulated, future studies are needed to determine if this results in reduced skeletal muscle structural integrity and force transduction.

### Body Mass and Leak Respiration

The modeled estimate of molt metabolism based on loss of body mass (48.7 MJ day^–1^) was consistent with previous indirect respirometry measures from similarly sized NES (46.5 MJ day^–1^) (Worthy et al., 1992), and provided context for measures of skeletal muscle metabolic capacity and mitochondrial leak capacity. Uncoupling proteins in skeletal muscle mitochondria allow transmembrane proton leak resulting in uncoupling of the ETC-derived proton gradient from ATP synthesis. While this dissipation of the proton gradient is wasteful with respect to ATP synthesis, this “futile” proton cycling is thermogenic and reduces the production of reactive oxygen species and resulting oxidative damage (Busiello et al., 2015). When assessing *ex vivo* measures of leak respiration, it is important to note that mitochondrial leak through these uncoupling proteins may be regulated by intracellular signals including glutathionylation of uncoupling proteins such as UCP3 (Mailloux et al., 2011, 2013). Therefore, the measures of mitochondrial leak respiratory capacity in this study do not indicate actual *in vivo* mitochondrial leak respiration.

Although NES spend considerable time in frigid waters (4–6°C) (Hakoyama et al., 1994), their large body mass, thick insulative blubber, vasoconstriction, and vascular countercurrent heat exchange are well adapted to conserve heat. Metabolic studies suggest NES are thermoneutral in water and susceptible to overheating on land where they exhibit heat-loss behaviors (White and Odell, 1971; Noren, 2002). Combining estimates of skeletal muscle mass with even the lowest measure of skeletal muscle leak respiratory capacity in this study (13 pmol O_2_ mg^–1^ s^–1^ or 17.5 ml O_2_ kg^–1^ min^–1^) results in an estimated whole body skeletal muscle leak respiratory capacity of 46.8 MJ day^–1^, which alone is roughly equivalent to whole body molt metabolism of 48.7 MJ day^–1^ (Table 2). Using the average mass of seals in our study of 337 kg and calculated metabolic rate of free-ranging NES (106.5 kJ kg^–1^ day^–1^) (Maresh et al., 2014) results in an estimated field metabolic rate of 35.9 MJ day^–1^ suggesting that leak respiratory capacity in the muscle alone exceeds estimates of the field metabolic rate in active animals at sea. Additionally, these muscle leak respiration estimates are based on the lowest measure of leak capacity from this study, and other measures of leak capacity are roughly twice as high (L_Omy_; Figure 1A and Supplementary Table S2). Although some degree of thermogenesis is undoubtedly important for maintaining thermoneutrality in marine mammals residing in cold waters, it is unlikely this high level of leak is fully required for thermogenesis.

### Skeletal Muscle Respiration

Skeletal muscle aerobic capacity is increased in response to increased activity via both qualitative and quantitative mitochondrial acclimation (Jacobs and Lundby, 2013). Likewise, skeletal muscle unloading results in metabolic deconditioning signaled by decreased mitochondrial content, reduced aerobic capacity, and a loss of muscle mass in typical terrestrial mammal models (Calvani et al., 2013). In a recent human study, maximal skeletal muscle respiratory capacity was reduced by approximately 30% following 21 days of bedrest (Salvadego et al., 2018). Metabolic deconditioning can occur rapidly; unloaded mouse soleus muscle decreased maximal respiratory flux significantly after only 3 days which preceded significant loss of muscle mass (Trevino et al., 2019). This early reduction in respiratory flux coincided with reduced intramuscular lipid handling and occurred before any change in mitochondrial content suggesting a link between substrate utilization and metabolic capacity. Unlike typical models of metabolic deconditioning following prolonged skeletal muscle unloading, the NES in our study had no significant decrease in maximal respiratory measures of OXPHOS or ETS despite more than 30 days of fasting and reduced activity (Figure 1A).

In addition to reduced maximum respiratory flux as assessed by OXPHOS and ETS, metabolic adaptation to changes in skeletal muscle load can also manifest as changes to the relative and absolute contribution of individual components of the electron transport system. We assessed changes in these respiratory ratios for use as a measure of metabolic conditioning. Strength and endurance training has been shown to increase the ratio of OXPHOS to ETS (FCR or P/E) (Pesta and Gnaiger, 2012). A decrease in FCR could signify metabolic deconditioning, however, no decrease occurred in molting NES (Figure 1B).

As the primary entry points for electrons to the ETC, skeletal muscle mitochondrial CI appears more susceptible to changes in load than CII. Increased physical fitness with exercise training results in increased mitochondrial respiratory capacity through CI in horses (Votion et al., 2012), and in a recent human study, 12 weeks of resistance exercise increased mitochondrial CI abundance with no change in CII abundance (Porter et al., 2015). Hindlimb unloading in mice resulted in decreased mitochondrial protein for complex I, complex III, and complex V, with no significant decrease for complex II (Cannavino et al., 2014). In the current study, there was no significant change in the absolute respiratory flux supported by CI or CII (Figure 1A and Supplementary Table S2), however, the relative contribution of CI to total OXPHOS as measured by SCR_CI_ was significantly reduced, potentially indicating a slight metabolic deconditioning associated with CI respiratory flux (Figure 1B).

### Metabolic Regulation

#### Seal Substrate Metabolism

As marine predators, seals are adapted to a diet consisting of high protein and fat with little carbohydrate (Keith and Ortiz, 1989; Houser et al., 2012). As a result, seals rely primarily on lipid oxidation to meet metabolic demand even in a fed state (Davis, 1983), and the reliance on lipid oxidation is further elevated during fasting (Castellini and Rea, 1992). Captive gray seals exhibit a shifting respiratory quotient indicating increased reliance on lipid oxidation with fasting (Boily and Lavigne, 1995). In addition, numerous studies demonstrate that NES also increase reliance on lipid oxidation during fasting in both pups and adults (Castellini et al., 1987; Castellini and Rea, 1992; Noren et al., 2003; Houser et al., 2012; Champagne et al., 2013). The gene expression data in this study is consistent with the previously reported small but significant shift further favoring lipid oxidation during fasting, although the physiological significance of this shift is unknown.

#### Genes Downregulated for Glucose Metabolism

During fasting, NES exhibit low blood insulin levels, reduced insulin sensitivity, and elevated blood glucose levels (Costa and Ortiz, 1982; Houser et al., 2013) resulting in high plasma glucose availability but low cellular oxidation. Consistent with fasting-induced suppression of glucose oxidation and promotion of lipid as a metabolic substrate, Enrichr pathway analysis identified “glycolysis/gluconeogenesis” pathways as statistically downregulated with five identified downregulated genes (Figures 2B,C). Other notable downregulated genes related to transport and metabolism of glucose include 4 solute carriers (*SLC22A3*, *SLC43A1*, *SLC16A2*, *SLC03A1*) associated with the transport of glucose and other sugars as well as *SLC2A4RG*, a primary regulatory gene for the insulin-responsive skeletal muscle glucose transporter GLUT4 (*SLC2A4*) (Gasser et al., 2007).

#### Genes Upregulated for Lipid Metabolism

Although pathway analysis did not identify a specific pre-populated list of lipid metabolism genes as a significantly altered pathway, there were a number of key genes that are critical to the regulation of skeletal muscle lipid metabolism that were significantly upregulated. Notably upregulated was the gene *PNPLA2*, which codes for adipose triglyceride lipase (ATGL). ATGL acts as the primary initial lipase and rate limiting step in the breakdown of intramuscular triglycerides to free fatty acids for oxidation (Haemmerle et al., 2011), and skeletal muscle overexpression of ATGL is associated with increased lipolysis and thought to influence mitochondrial biogenesis and oxidative capacity (Morales et al., 2017). The gene coding for the β2-adrenergic receptor (*ADRB2*) was also upregulated. Increased ADRB2 signaling in skeletal muscle is associated with slow oxidative endurance-type performance by suppressing glycolysis and promoting lipid oxidation (Sarpeshkar and Bentley, 2010). Also upregulated was pyruvate dehydrogenase kinase (*PDK4*), a primary metabolic regulator that inhibits glucose oxidation to shift metabolic pathways toward fatty acid oxidation (Fritzen et al., 2015). Upregulation of PDK4 is critical to prioritize lipid oxidation and spare carbohydrate in hibernating animals (Morin and Storey, 2009). Also of note is upregulation of the regulator of lipid metabolism, lipin 1 (*LPIN1*) (Finck et al., 2006; Rashid et al., 2019). Skeletal muscle overexpression of lipin 1 is associated with decreased insulin sensitivity (Phan and Reue, 2005) as seen in fasting NES (Houser et al., 2013). The upregulated *NFE2L1* gene (aka Nrf1) also regulates lipid metabolism (Kim et al., 2016), as well as skeletal muscle mitochondrial biogenesis and respiratory capacity (Hoppeler and Flück, 2003). The upregulated *OSBPL10* gene codes for a member of the oxysterol-binding proteins, a family of proteins which play diverse roles in regulating the intracellular metabolism, transport, and signaling of lipids (Fairn and McMaster, 2008).

#### HIF-1 Alteration

Significant downregulation in the “HIF-1 signaling pathway” by Enrichr pathway analysis suggests a decrease in hypoxic signaling in NES skeletal muscle during molt. In addition to diving apneas, NES also have regular apneustic periods on land (e.g., resting sleep apneas). These breath holding periods are associated with bradycardia, reduced skeletal muscle blood flow, and myoglobin oxygen desaturation, but this resting skeletal muscle is not under the same locomotor metabolic demands as diving (Ponganis et al., 2008). The postweaning fasting period in newborn NES pups is contrastingly associated with increased HIF gene expression (Soñanez-Organis et al., 2013). However, the postweaning fasting period represents a developmental phase where the skeletal muscle is distinct from that of adults (Noren et al., 2001; Chicco et al., 2014; Moore et al., 2014), and the pups are not conditioned to repeated and prolonged active breath hold dives as molting adults are. Given the transition from an active lifestyle of repeated breath hold diving to a sedentary terrestrial period seen in the molting adult NES in our study, it is unsurprising that gene expression associated with hypoxic signaling is reduced during this period. It should also be noted that HIF-1 activation leads to suppressed aerobic lipid oxidation in favor of anaerobic glycolytic pathways (Kim et al., 2006). Downregulation of hypoxic signaling is consistent with promotion of pathways favoring aerobic lipid-based metabolism.

#### Circadian Regulation

Peripheral circadian cycles of gene expression regulate diurnal metabolic cycles for transitioning from active periods of greater glucose oxidation to resting periods favoring fat oxidation in mice (Dyar et al., 2014), and are prominent in regulating mitochondrial metabolism and substrate utilization (Ezagouri and Asher, 2018). Skeletal muscle circadian clocks respond to cyclic stimuli including feeding and activity through regulatory genes including *CLOCK*, *ARNTL* (aka *BMAL1*), and the period-family circadian regulator *PER1* (Schroder and Esser, 2013). Periods of fasting have been shown to alter the expression of key skeletal muscle circadian regulators and the cyclic expression of functional genes in fish skeletal muscle (Wu et al., 2018). In the current study, key circadian rhythm related genes were upregulated during molt including *ARNTL* and *PER1* (Figure 2D), and these genes are both tied to circadian regulation of mitochondrial lipid metabolism (Ezagouri and Asher, 2018). In addition, *USP2* was upregulated which de-ubiquitinates PER1 thereby extending its cellular activity. Skeletal muscle *CLOCK* regulated circadian cycles are also closely tied to HIF-1 regulation (Peek et al., 2017). Although the etiology and mechanisms of how these circadian cycles, HIF-1 pathways, and lipid metabolism pathways converge, the altered gene expression in these related pathways is noteworthy given these recent studies.

### Fasting-Induced Metabolic Changes May Protect Muscle From Unloading

Although decreased activity and reduced skeletal muscle load typically results in loss of muscle mass and metabolic capacity (Seene et al., 2012; Frontera and Ochala, 2015; Mukund and Subramaniam, 2019), muscle is largely preserved in both molting NES (Worthy et al., 1992) and hibernating mammals (Cotton, 2016). Commonalities between these unique animal models could provide novel insights into pathways that regulate skeletal muscle to explain how their muscle is preserved despite lengthy periods of reduced load. Both hibernating mammals and molting NES experience prolonged periods of nutrient deprivation resulting in a shift in skeletal muscle metabolic substrate utilization toward mobilizing fat stores for lipid oxidation (Castellini and Rea, 1992; Buck et al., 2002; Vermillion et al., 2015; Cotton, 2016). Within skeletal muscle, fasting-induced nutrient deprivation results in upregulation of peroxisome proliferator-activated receptor γ coactivator-1α (PGC-1α) (Finck et al., 2006; Gerhart-Hines et al., 2007; Hempenstall et al., 2012; Hatazawa et al., 2018), which can be upregulated in response to either reduced intracellular glucose oxidation (Gerhart-Hines et al., 2007) or increased lipid signaling (Haemmerle et al., 2011; Nakamura et al., 2014; Biswas et al., 2016). PGC-1α is a master regulator of mitochondrial biogenesis and metabolism in type 1 aerobic skeletal muscle, and also protects against disuse muscle atrophy (Liang and Ward, 2006; Kang and Li Ji, 2012). By stimulating PGC-1α pathways, fasting-induced shifts in metabolic substrate favoring fatty acid oxidation may help to protect skeletal muscle from the detrimental effects of unloading as proposed in hibernating mammals (Cotton, 2016). Through this mechanism, fasting-induced metabolic substrate shifts in NES may help preserve the mass and metabolic capacity of slow oxidative fibers in the absence of exercise-induced stimuli, thereby facilitating the rapid return to foraging and activity following the extended periods of reduced activity. In addition, this selective preservation of type 1 skeletal muscle fibers during repeated bouts of prolonged fasting may also explain the unique ontogenetic transition in NES which have type II fast twitch fibers present in pups but essentially absent in adults (Moore et al., 2014). By better understanding the adaptive mechanisms in these unique animal models, we may expose metabolic pathways that can be manipulated to manage loss of muscle mass and respiratory capacity associated with aging, disease, and reduced muscle load.

## Summary

Previous studies have shown that NES skeletal muscle mass is largely preserved during the annual molt despite more than a month of fasting and reduced activity. In the current study, we determined that skeletal muscle metabolic capacity is also preserved, although decreased expression of collagen and ECM components may indicate loss of skeletal muscle structural integrity and force transduction. Leak respiratory capacity in skeletal muscle alone appears more than adequate to meet the thermogenic demands in adult NES. We found altered expression of key regulatory genes in skeletal muscle over the course of the molt indicating: (a) altered circadian regulation of metabolism Fasting-induced metabolic shifts promoting lipid oxidation in NES skeletal muscle may play a role in preserving the mass and metabolic capacity of type I muscle fibers despite unloading and deprived nutrient intake, and facilitate a rapid return to activity.

## Data Availability Statement

Gene expression data has been deposited in NCBI’s Gene Expression Omnibus (Edgar et al., 2002) and is accessible by GEO accession number GSE151509 (https://www.ncbi.nlm.nih.gov/geo/query/acc.cgi?acc=GSE151509).

## Ethics Statement

The animal study was reviewed and approved by the Animal Care and Use Committee of the University of California, Santa Cruz.

## Author Contributions

RD, DC, CP, MS-M, and TW conceived and designed the study. RH, LH, CD, CP, SW, and TW performed the data collection. RD, DC, TW, and MS-M facilitated. RD, CP, SW, MS-M, and TW conducted the data analysis and interpretation. TW drafted the manuscript with critical revisions by RD, RH, LH, SW, TW, and MS-M. All authors viewed and approved the final manuscript.

## Conflict of Interest

The authors declare that the research was conducted in the absence of any commercial or financial relationships that could be construed as a potential conflict of interest.

## Footnotes

1 https://www.ncbi.nlm.nih.gov/geo/query/acc.cgi?acc=GSE151509

## Abbreviations

NES: northern elephant seal
ECM: extracellular matrix
FC: fold change
PGC-1α: peroxisome proliferator-activated receptor γ coactivator-1 α
SUIT: substrate uncoupler inhibitor titration
OXPHOS: oxidative phosphorylation (respiratory capacity)
L_n_: leak respiration measured with substrates and no adenylates
L_Omy_: leak respiration measured with inhibition of ATP synthase
ETS: electron transport system (respiratory capacity)
CI: mitochondrial respiratory complex I (respiratory capacity)
CII: mitochondrial respiratory complex II (respiratory capacity)
ADP: adenosine diphosphate
SCR: substrate control ratio (respiratory ratio of CI/OXPHOS)
CCR: coupling control ratio (respiratory ratio of L_n_/OXPHOS)
FCR: flux control ratio (respiratory ratio of OXPHOS/ETS)
cGMP: cyclic guanosine monophosphate
PKG: protein kinase G (cGMP-dependent protein kinase)
KEGG: Kyoto encyclopedia of genes and genomes
ATGL: adipose triglyceride lipase.
