# Supplementary material for: Changes in Northern Elephant Seal Skeletal Muscle Following Thirty Days of Fasting and Reduced Activity
Source: Front Physiol. 2020 Oct 6;11:564555. doi: 10.3389/fphys.2020.564555 (PMC7573231; doi:10.3389/fphys.2020.564555)

Supplementary Material

**Supplemental Table S1.** Summary of northern elephant seal skeletal muscle RNAseq read mapping aligned with Hawaiian monk seal reference genome.

| **Sample** | **Total Read Pairs** | **Uniquely mapped (%)** | **Multi-**  **Mapped (%)** | **Unmapped (%)** |
| --- | --- | --- | --- | --- |
| 4448B | 97101964 | 78.5 | 4.5 | 17 |
| 5572A | 71708646 | 79 | 4.8 | 16.2 |
| 5572B | 54500422 | 79.6 | 4 | 16.4 |
| 5842A | 63160954 | 72.5 | 5.3 | 22.2 |
| 6762A | 83533277 | 76.3 | 4.7 | 19 |
| 9678B | 60421471 | 64.5 | 3.8 | 31.7 |
| C497A | 89559104 | 76.3 | 4.4 | 19.3 |
| X410B | 73056612 | 78.4 | 4.3 | 17.3 |

**Supplementary Table S2.** Average northern elephant seal skeletal muscle respiratory rates (pmol O_2_ mg^-1^ s^-1^) and ratios (SD) before and after approximately one month of haul-out during yearly molt. Statistical significance (p < 0.05) is denoted with *.

|  | **Pre-molt** | **Post-molt** | **p-value** |
| --- | --- | --- | --- |
| **Respiratoy Rate** | |  |  |
| **L_n_** | 13.0 (6.1) | 14.5 (2.0) | 0.602 |
| **L_Omy_** | 28.2 (11.4) | 24.1 (6.4) | 0.298 |
| **OXPHOS** | 100.5 (36.9) | 97.6 (17.1) | 0.753 |
| **ETS** | 116.5 (45.4) | 108.9 (20.1) | 0.553 |
| **CI** | 74.0 (35.2) | 59.8 (16.1) | 0.219 |
| **CII** | 95.9 (37.2) | 91.3 (15.7) | 0.662 |
| **Respiratory Ratio** | |  |  |
| **CCR_Ln_** | 0.13 (0.03) | 0.15 (0.03) | 0.181 |
| **FCR** | 0.87 (0.03) | 0.90 (0.02) | 0.085 |
| **SCR_CI_** | 0.72 (0.09) | 0.61 (0.08) | 0.033* |
| **SCR_CII_** | 0.95 (0.06) | 0.94 (0.02) | 0.756 |

**Supplementary Table S3a.** Downregulated genes in northern elephant seal skeletal muscle following one month of reduced activity and fasting during the annual molt. Genes were identified as downregulated if they expressed significantly differential expression in the two conditions (pre / post molt) (P_FDR_ < 0.05) with a log_2_ fold change of less than -1 (172 downregulated genes).


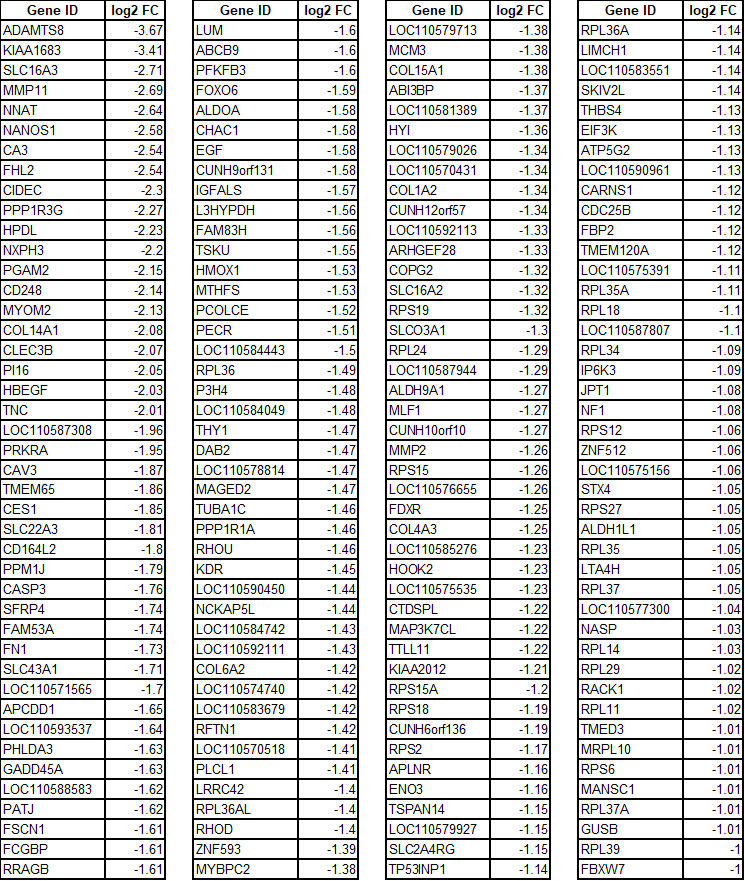


**Supplementary Table S3b.** Upregulated genes in northern elephant seal skeletal muscle following one month of reduced activity and fasting during the annual molt. Genes were identified as upregulated if they expressed significantly differential expression in the two conditions (pre / post molt) (P_FDR_ < 0.05) with a log_2_ fold change of greater than 1 (162 upregulated genes).


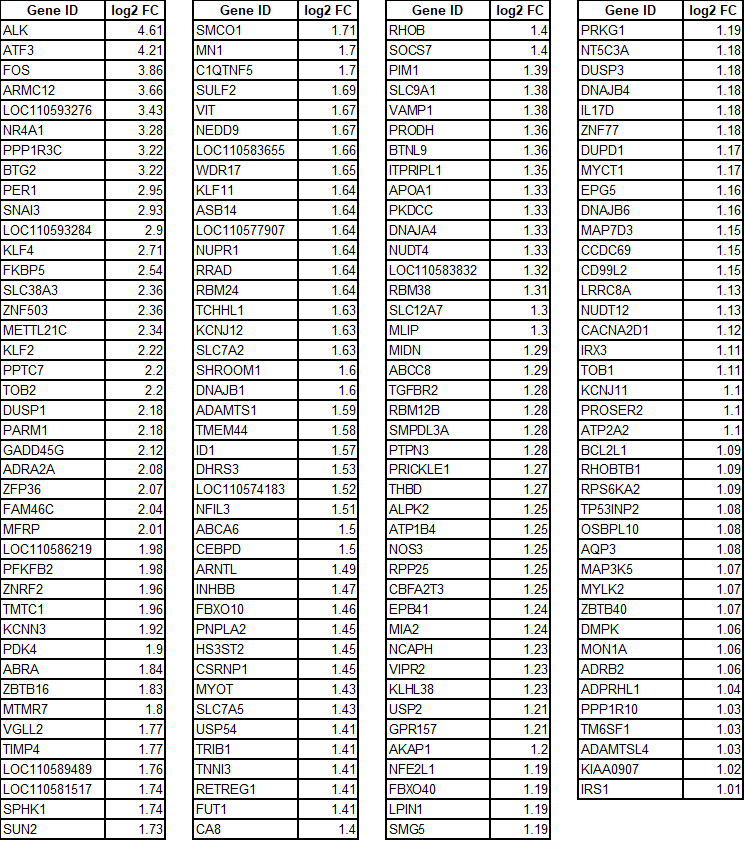

Supplement: Supplementary file 1 [file Table_1.DOCX]
